# Rhizosphere microbiomes of field-grown *B. stricta* exhibit minimal diel changes in microbial membership and protein synthesis potential

**DOI:** 10.3389/fmicb.2025.1609057

**Published:** 2025-09-25

**Authors:** Alessandra Ceretto, Cynthia Weinig

**Affiliations:** ^1^Department of Botany, University of Wyoming, Laramie, WY, United States; ^2^Program in Ecology, University of Wyoming, Laramie, WY, United States; ^3^Department of Molecular Biology, University of Wyoming, Laramie, WY, United States

**Keywords:** rRNA (ribosomal RNA), 16S rRNA (16S rDNA), plant circadian clock, rhizosphere bacteria, host microbiome interaction

## Abstract

The rhizosphere microbiome has a significant impact on plant health and fitness. Quantifying bacterial responses to fine-scale plant-mediated changes in the rhizosphere, such as those associated with diel cycling in host plant physiology, will increase our understanding of microbial community assembly patterns. Here, we used 16S rRNA biomarker gene (DNA) and transcript (RNA) sequencing to characterize changes in the rhizosphere community membership and PSP over short timescales in field-grown *Boechera stricta* (*B. stricta*) plants. Microbial communities characterized by 16S-rRNA-transcripts, which serve as a proxy for microbial protein synthesis potential (PSP), showed greater sensitivity to fine-scale environmental changes than did communities characterized by 16S-rRNA biomarker gene sequencing, which reflects microbial presence/absence. Significant differences were observed between communities characterized by RNA vs. DNA, with RNA-derived communities showing greater alpha and beta diversity differences between the rhizosphere vs. control soil communities within phyla and in differential abundance analysis of genera. Communities reconstructed from RNA were more sensitive to the effects of field blocks and collection timepoints. Differential abundance analysis revealed significant differences (*p* < 0.05) between the pre-dawn (AM) and early afternoon (PM) timepoints for five genera based on 16S rRNA transcripts. This included the plant-associated genus Curtobacterium. However, when variance was partitioned between days of collection, the amplitude of the signal between diel timepoints was non-significant. In summary, community composition and protein synthesis potential were highly sensitive to abiotic factors expressed over the small spatial scale of field blocks and short 24-h periods between collection days but showed minimal to no diel patterning.

## Introduction

Microorganisms that reside in the thin layer of soil around a plant's roots, known as the rhizosphere, affect plant growth, health, and fitness in a variety of direct and indirect ways (Bashan, 1998; Henning et al., 2016; Lugtenberg and Kamilova, 2009; Zarraonaindia et al., 2015). Rhizosphere microorganisms, called the rhizosphere microbiome, can relieve and increase plant tolerance to abiotic stressors (Mendes et al., 2013; Zolla et al., 2013), provide a plant protection against disease (Mendes et al., 2011; van der Voort et al., 2016), increase plant access to nutrients (Chen et al., 2014; Mendes et al., 2013; Richardson and Simpson, 2011), and can even alter plant phenology, such as flowering time (Panke-Buisse et al., 2015; Wagner and Mitchell-Olds, 2018).

The rhizosphere microbiome is sensitive to discrete chemical changes in the soil environment, some of which are mediated by the plant, such as the alteration of the rhizosphere chemical environment via root exudates (Bulgarelli et al., 2013; Mendes et al., 2013; Philippot et al., 2013; van der Voort et al., 2016; Wagner and Mitchell-Olds, 2018; Zarraonaindia et al., 2015) and drying-rewetting cycles and other changes in water potential in the rhizosphere (Cheng, 2009; Montaldo and Oren, 2022). Much of plant physiology and metabolism exhibits diel cycles in activity, reflecting either direct responses to daily changes in light levels or other abiotic factors or the effects of endogenous circadian clock activity (Nobs et al., 2019; Seaton et al., 2015). Beyond its effects on plant physiology, the plant circadian clock has been shown to influence the rhizosphere microbial community composition in controlled settings (Baraniya et al., 2018; Hubbard et al., 2023, 2018; Staley et al., 2017; Zhao et al., 2021). The plant circadian clock may modulate the rhizosphere microbiome via determination of the timing, composition, and release of root exudates (Hubbard et al., 2023; Li et al., 2021; Wang et al., 2020; Zhao et al., 2021) as well as the timing of drying-rewetting cycles in the rhizosphere (Matimati et al., 2014). Microorganisms can possess their own circadian clock, which can respond to biotic cues from host plants as well as abiotic diurnal cues in the bulk soil environment (Johnson et al., 2017; Kearns et al., 2017; Vandecar et al., 2009).

To better understand the nuanced and time-sensitive compositional changes in the microbial community over short time scales, this study uses 16S rRNA-transcript to characterize microbial community protein synthesis potential (PSP) in addition to using 16S rRNA-gene community characterization. The 16S rRNA-transcript (referred to hereafter as RNA) is essential to protein synthesis and is the non-coding nucleic acid component of the small ribosomal subunit (Lindahl, 1975; Nomura et al., 1984); the 16S rRNA-gene (referred to hereafter as DNA) is the conserved marker gene, which encodes the small ribosomal subunit (Caporaso et al., 2012; Kozich et al., 2013). RNA reflects a microbial population's potential to synthesize proteins via the presence of ribosomes, or protein synthesis potential (PSP; Blazewicz et al., 2013), rather than the total microbial membership of a community shown by DNA, which can reflect the presence of DNA from dead or lysed cells, extracellular free DNA, and dormant cells that may not be significantly active within a community (Bakken and Frostegård, 2006; England et al., 2004; Hamilton et al., 1968; Lorenz and Wackernagel, 1987). Bacterial rRNA degrades more quickly than DNA, sometimes even within hours of being synthesized, depending on abiotic factors and the health of the cell (Karl and Bailiff, 1989; Schostag et al., 2020; Zundel et al., 2009), and thus provides finer-scale temporal resolution of the microbial community's composition. Furthermore, an RNA-derived microbial community is more sensitive to ambient conditions than a DNA-derived microbial community, as the cell increases or decreases the number of ribosomes inside it to respond to the environmental changes (Charvet et al., 2014; De Vrieze et al., 2018; Hunt et al., 2013; Li et al., 2019). The greater sensitivity to both time and slight changes in the soil environment provided by the RNA-derived microbial community likely will reveal more than only DNA about the small time-scale changes in the rhizosphere microbial community driven by the plant's diel cycling.

While several recent studies using controlled growth environments and experimental plant genotypes with a mistimed circadian clock have shown that microbial community composition is sensitive to the host plant clock (Harmer, 2009; Hubbard et al., 2023, 2018; Maddur Puttaswamy, 2019; Staley et al., 2017; Zhao et al., 2021), the agroecological relevance of this effect has yet to be tested in a field setting. Using the short-lived perennial, *Boechera stricta* (*B. stricta*), grown in its native sites, this study characterizes microbial community dynamics over short diel timescales. We characterized microbial membership using 16S DNA as well as via 16S rRNA, the latter of which is a more sensitive biomarker of change in the microbial community over short timescales due to its comparatively lower stability. Plants and bulk soil controls were collected at a pre-dawn and early afternoon timepoints over the course of 3 days. We hypothesized (1) that bacteria enriched in the rhizosphere would show day-night changes in PSP and total microbial membership that are distinct from those observed in the control soils, and (2) that RNA would show more dynamic changes than DNA due to its greater sensitivity to fine-scale environmental changes, such as those that occur in the rhizosphere due to top-down plant-driven changes in the rhizosphere environment.

## Methods

### Plant material and growth conditions

Seeds of a Wyoming native perennial herb, *B. stricta*, were originally collected from the Snowy Range Mountains (41.32971759902109 N, −106.50515422710646 W) and grown for one generation in the greenhouse to increase seed numbers and minimize maternal effects. Before planting, seeds were surface sterilized by rinsing for 1 min in a mixture of 70% ethanol, 0.1% Triton, and 30% RO water, followed by rinsing in RO water, and then rinsing for 12 min in a mixture of 10% bleach, 0.1% Triton, and 90% water. Seeds were then rinsed three more times with RO water before being placed on sterile filter paper for ease of planting (adapted from Lundberg et al., 2012).

Field soil was collected from unvegetated sites adjacent to a field location with a native *B*. *stricta* population, referred to hereafter as the Crow Creek field site (CRW), and sieved to 4 mm to remove large debris. The soil was then autoclaved three times for 30 min, with mixing between autoclaving steps. The soil was then mixed with autoclaved potting soil [Redi-Earth Potting Mix (Sungro Horticulture, Agawam, MA, USA) in a 9:1 ratio]. We included this small percentage of potting mix because its greater water-holding capacity relative to the field soil improves the overall rate and synchrony of seed germination. This soil mixture was next inoculated with a 4% v/v of non-autoclaved field soil inoculum. Although autoclaving reduces the similarity of the study system to the natural field soil environment, it reduces variability between replicates and removes any pest organisms within the soil. Inoculum from field soil was added to restore the autoclaved soil to a similar but not identical microbial soil ecosystem.

This soil mixture was used to plant the surface-sterilized seeds. In addition to pots planted with seeds, which would be used to characterize the rhizosphere microbiome, we prepared soil-filled pots without plants, which would be used to estimate the microbiome of bulk soil or unvegetated microsites, referred to hereafter as the “control soil.” Seeds were germinated in 2″ mesh net pots (2″ Inch TEKU Net Slit Pots for Hydroponic Aeroponic Use) with the bottom surrounded by sterilized coffee filters and plastic cups to prevent soil erosion. Plants were allowed to grow for 4 weeks under greenhouse conditions (UW Laramie Research and Extension Center, Laramie, WY) with ambient day/night light and temperature cycles before field transplanting. Mesh pots were placed in a randomized checkerboard array in tray blocks, such that no pot was directly adjacent to another. Plastic covers were placed over all trays to retain humidity and promote germination. All materials for planting, such as bench tops, trays, pots, and covers, were bleached and rinsed before use. Pots were individually watered, initially via subirrigation and after 2 weeks of growth via overhead misting. Covers were removed 2 weeks after germination. Three weeks after germination, all pots were acclimated to the outside environment via 2-h field exposures.

Four weeks after plant germination in May of 2022, plants were transferred to the Crow Creek field site, which has a naturally occurring population of *B. stricta*. The CRW study site is located in the Medicine Bow-Routt National Forest in southeastern Wyoming (41.227318 N, −105.383343 W), at an elevation of ~2,560 m. Six 26 cm × 140 cm plots were cleared of plants and debris. Mesh pots were randomly assigned to each plot and planted approximately 10 cm apart in two rows of 24 pots. Mesh pots were removed from filters and cups and placed directly in field site plots to minimize root damage while transplanting and to facilitate the collection of plant rhizospheres. Plant germination time, rosette size, and true leaf number were measured weekly to estimate plant performance. All pots were surface watered every other day using RO water and checked for insect damage.

### Sample collection and processing

**Figure 1:**
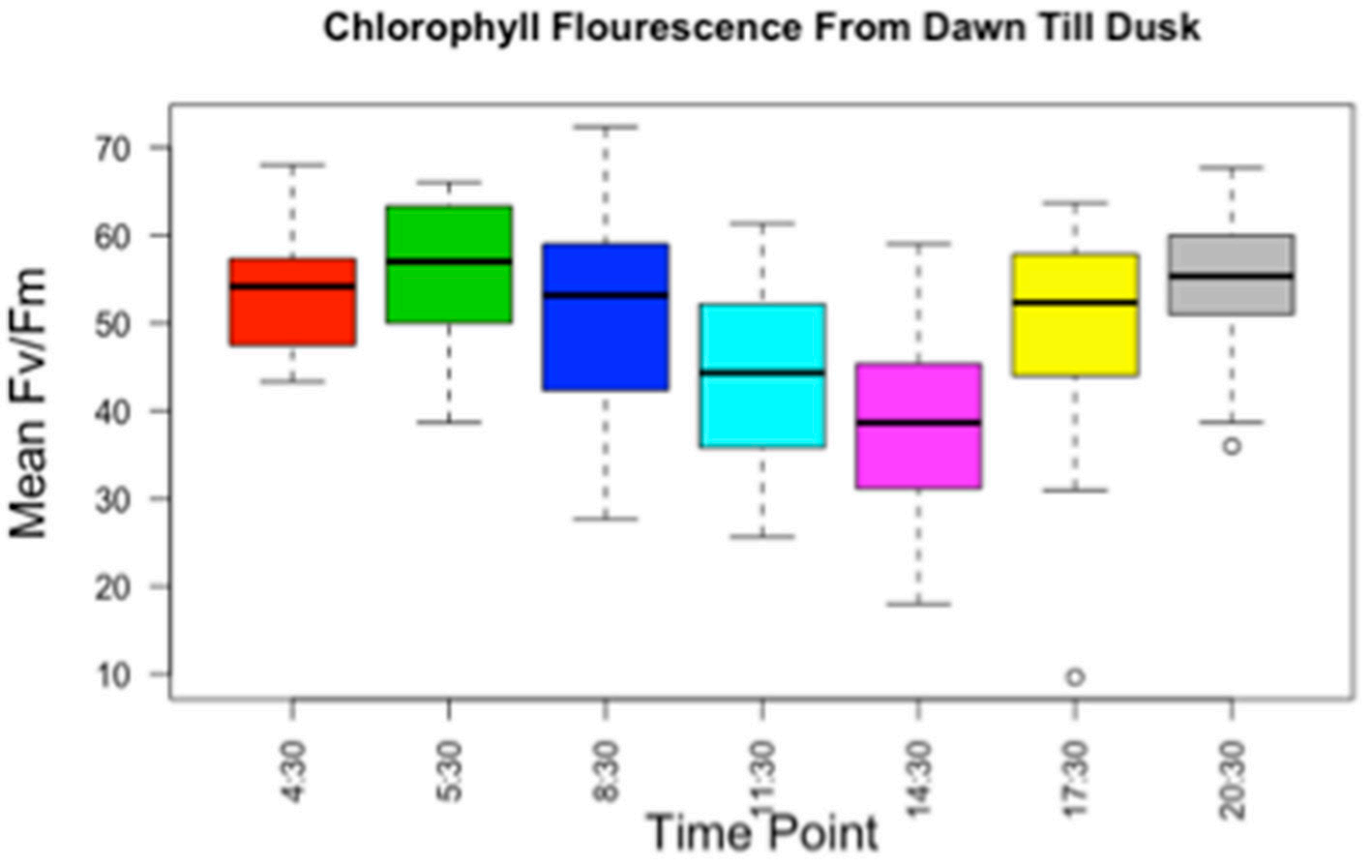
Chlorophyll fluorescence of *B. stricta* from dawn till dusk. The *Y*-axis is mean Fv′/Fm′, and the *X*-axis is timepoint sampled, from pre-dawn 4:30 AM to after sunset 8:30 PM (20:30). Lines in the boxes indicate the median, with the top and bottom of boxes representing 75th and 25th quartiles. Whiskers represent the 1.5 × interquartile range (IQR).

Before planting in the field, while the plants were acclimating to the environment outside the greenhouse, we collected chlorophyll fluorescence data as a measure of photosystem II activity (Murchie and Lawson, 2013) over 5 days. Specifically, for each plant, we measured chlorophyll fluorescence every 3 h starting at 4:30 AM before sunrise, with an additional timepoint at 5:30 AM immediately after sunrise, and continuing until 8:30 PM after sunset. This data was used to choose two timepoints where the inferred physiological activity of the plant was most divergent from one another, which we hypothesized could reflect timepoints when the microbial community was most divergent if plant physiology were affecting the rhizosphere environment. We selected a pre-dawn timepoint of 3 AM and an early afternoon timepoint of 2 PM (Figure 1).

Four weeks after transplanting the plants to the field, when the plants were 8 weeks past germination, soil and plant samples were harvested over three consecutive days, from June 22 to June 24, 2022. Sampling occurred during pre-dawn hours from 3:00 to 3:30 AM and in the early afternoon from 2:00 to 2:30 PM. Samples were randomly collected, ensuring an equal number of samples per treatment of rhizosphere and control soils, with samples taken at both AM and PM. Samples from the two treatment types were handled as follows: (1) For the pots without plants, soil was stored in whirl packs (Whirl-Pak^®^ Standard Sterilized Sampling Bags, Nasco Sampling LLC., Pleasant Prairie, WI, United States), and smaller subsamples of these soils were taken and stored in 2 mL test tubes for later nucleic acid extraction; (2) For collection of rhizosphere soil from the pots with plants, mesh pots were removed from the soil. Care was taken to ensure that roots growing out of the mesh pots were damaged minimally; plants were then removed from the pots and shaken to remove excess loose soil. Soil that adhered closely at approximately 1 mm to the root mass was considered rhizosphere soil. Roots and adhered soil were separated from plant leaves and stems using flame-sterilized scissors and placed in Falcon tubes containing approximately 200 mL of PBS buffer (200 μL Silwet, 900 mL RO water, 100 mL 10 × PBS; adapted from Bulgarelli et al., 2012). Samples were stored on ice during the fieldwork. Immediately upon returning to the lab, the control soil samples were transferred to a −80 **°**F freezer, and the rhizosphere soil samples were processed. Falcon tubes containing rhizosphere soil and plant root tissue were defrosted, then lightly vortexed to remove adhered soil from root tissue. Root tissue was removed using sterile forceps. The soil slurry was then vacuum-filtered through a 0.2 nm filter. The resulting soil and filter were transferred to a 0.5 mL centrifuge tube and flash-frozen with liquid nitrogen before being stored long-term at −80 °C.

### Nucleic acid extraction and amplicon library preparation

This study sought to eliminate biases that might artificially inflate the differences between DNA- and RNA-derived community composition measurements. Methodological biases were minimized via the simultaneous extraction of nucleic acids from a single soil sample, so only one community profile from one soil aliquot in both DNA and RNA was analyzed (Gentile et al., 2006; McCarthy et al., 2015; Moeseneder et al., 2005; Morgan et al., 2010). Different methods of RNA and DNA extraction, such as RNA having a reverse transcription step, whereas DNA does not, cannot be corrected for (Zhen et al., 2015). Control and rhizosphere soil RNA and DNA samples were simultaneously extracted using the methods described in the RNeasy PowerSoil Total RNA Kit and RNeasy PowerSoil DNA Elution Kit (https://digitalinsights.qiagen.com). After extraction, RNA was reverse-transcribed into cDNA using the QuantiTect Reverse Transcription Kit (https://digitalinsights.qiagen.com). Negative control blank samples were included for extractions and reverse transcription. Samples were stored at −20 °C until further processing.

Both 16S-rRNA-gene (DNA) and 16S-rRNA-transcript (RNA) amplicons were sent to The University of Chicago Marine Biological Laboratory in Woods Hole for sequencing and taxonomic identification (https://vamps.mbl.edu). Illumina MiSeq was used with bacterial v4v5 primers, and taxonomy was assigned using the SILVA 119 database on the VAMPS (Visualization and Analysis Microbial Population Structures) website (Huse et al., 2014). The SILVA 119 database was used so that a direct comparison of taxa between this and previous studies (i.e., Ceretto and Weinig, 2025) would be equivalent.

### Bioinformatics and data analysis

Sequences, read counts, taxonomic IDs, and sequence ID names were downloaded from the MBL VAMPS website portal (Huse et al., 2014) for OTU table generation and analysis. RNA samples underwent an extra round of PCR compared to DNA samples, as cDNA is single-stranded and requires an extra step to compare with double-stranded DNA.

Reads that were not assigned to the kingdom of bacteria were removed. Reads that appeared in the negative control samples and were present at low frequency in other samples (i.e., less than 100 reads per sample) were considered contamination and subsequently removed. Sequences that occurred five or more times in all samples were considered potential OTUs. Samples with less than 25,000 total reads were removed from analysis, and samples were filtered so each RNA-derived sample had a corresponding DNA sample that was extracted from the same collected soil. OTUs that occurred in less than 5% of all samples with less than two reads were removed.

The final data set consisted of 234 total samples: 117 RNA samples, comprising 63 from rhizosphere soil and 54 from control soil, and 117 DNA samples, comprising 63 from rhizosphere soil and 54 from control soil. These samples contained 11,402 unique OTUs and 11,137,182 total reads (63% RNA reads and 36% DNA reads). Notably, selecting an even number of samples at random from each treatment rhizosphere, or control, did not alter the conclusions of the analysis; therefore, we present results based on all 234 samples.

In R [version 4.3.1 (2023-06-16)], samples were rarefied to 25,491 reads per sample, and alpha diversity was estimated using the phyloseq package “estimate_richness,” with differences between alpha diversity categories determined using Student's *T*-test. Statistical differences between community nucleic acid derivation and soil type were determined using ANOVA (analysis of variance). To calculate differences between the RNA- and DNA-derived communities, the model OTU abundance (within the RNA- or DNA-derived community) = Soil Type ^*^ Collection Date ^*^ Field Block, where soil types were rhizosphere and bulk-control soils, collection date was 1 of 3 days where samples were collected, and field block is the approximately two by 1.0 m rectangular field block at the CRW site which contained study samples. The model Soil Type ^*^ Collection Date ^*^ Field Block was used to calculate significance for the RNA- and DNA-derived communities separately.

We calculated the significance of the collection timepoint (AM or PM). To remove the confounding effect of collection date on collection time, samples were first separated into three groups based on the date of collection and whether they were derived from RNA- or DNA-derived communities. The model OTU abundance (within the RNA- or DNA-derived community) = Soil Type ^*^ Collection Time ^*^ Field Block was used to calculate significance, where Collection Time represented the AM and PM timepoints. Samples were separated by RNA- and DNA-derived communities and soil type, but kept together based on collection day to calculate significance again using the model OTU abundance (within RNA- or DNA-derived community) = Collection Time.

To calculate beta diversity, we used the package Phyloseq v1.30.0 (McMurdie and Holmes, 2013). Reads assigned to an OTU in a sample were divided by the total number of reads per sample to calculate within-site proportional abundances. PCA plots were used to visualize Bray-Curtis pairwise dissimilarities in community data. Significant differences were detected between RNA- and DNA-derived communities, as well as between the rhizosphere and control soils within and between communities using PERMANOVA (pairwise Adonis testing with Bonferroni correction). Barplots comparing taxa within the samples were generated using the proportional abundances of reads, with pairwise *t*-tests used to determine significance between groups. The corncob package (Martin et al., 2020) was used to estimate differential abundances between the RNA- and DNA-derived communities, as well as between the RNA-derived and DNA-derived rhizosphere and control soil samples, and between the RNA-derived and DNA-derived rhizosphere and control soil samples by collection time of 3 AM and 2 PM, using the absolute abundance of reads, rather than proportional abundances.

## Results

### Timepoint selection

Chlorophyll fluorescence data showed a change in mean Fv′/Fm′ from dawn to dusk, with the lowest mean Fv′/Fm′ around 2 PM. A high mean Fv′/Fm′ was observed at timepoints when it was dark, pre-dawn at 4:30 AM and post-sunset at 8:30 PM (Figure 1). Although the highest mean Fv′/Fm′ occurred at 5:30 AM just after sunrise, we chose the pre-dawn timepoint, because it provided a large window of time to collect samples in the dark instead of rushing to finish sampling during the brief hour of dawn. It also allowed us to collect samples earlier, before sunrise, when it was still dark, if more time was needed for collection. We chose not to collect samples immediately after sunset, as we felt giving the plants longer to acclimatize to night conditions would better magnify any diel changes mediated by the plant in the rhizosphere community.

### Differences in community composition vs. protein synthesis potential based on DNA vs. RNA community reconstruction

Between the RNA- and DNA-derived communities, some phantom taxa and reads from possibly inactive or dead cells were detected but made up less than 1% of the total number of reads. After data QA/QC, 394 taxa, represented by 1,018 reads, were unique to the RNA-derived community profile, and 44 taxa, consisting of 222 reads, were unique to the DNA-derived community.

**Figure 2:**
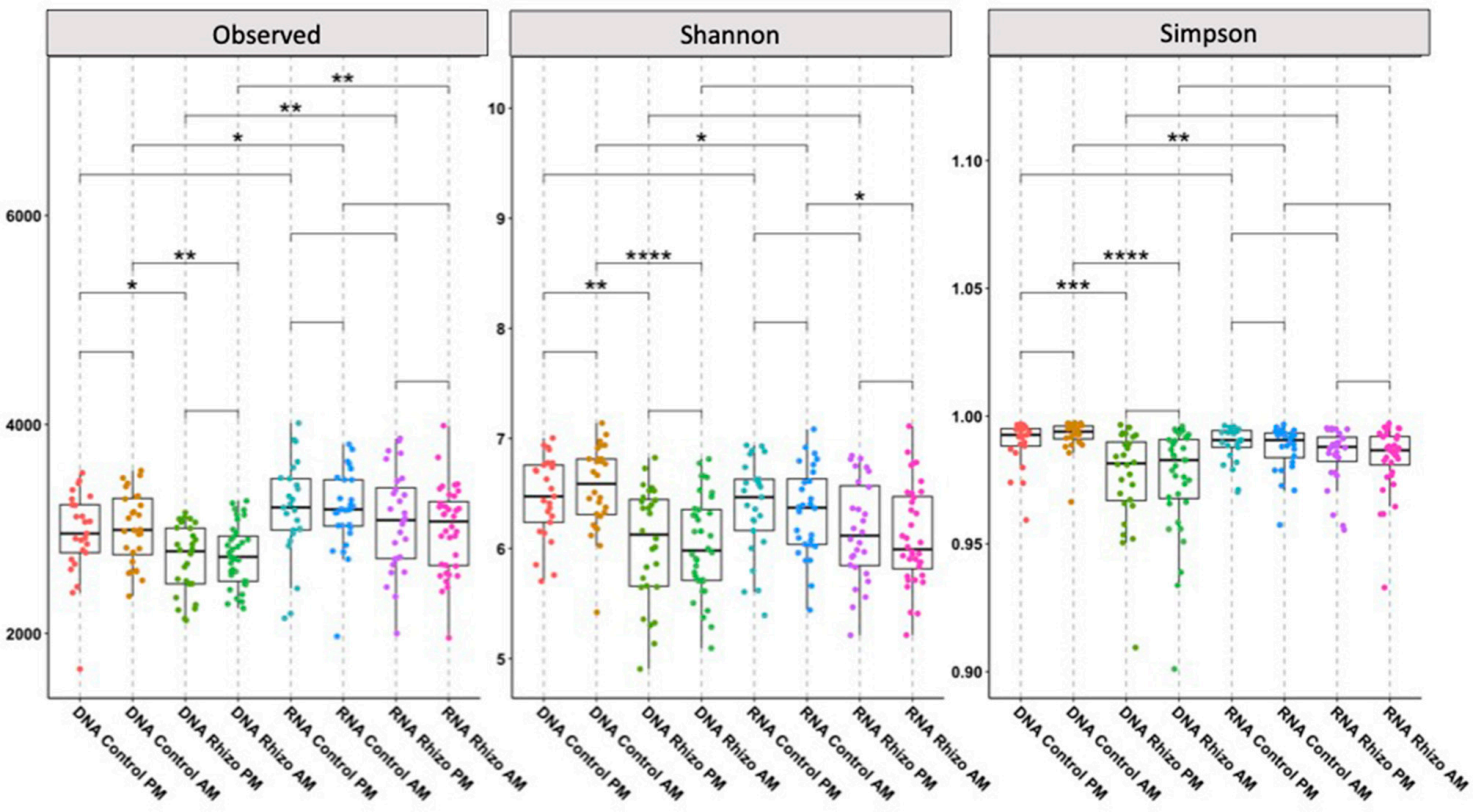
Alpha-diversity metrics for DNA- and RNA-derived community soil type and time collection comparison. Columns indicate the community being investigated (DNA-derived soil types at AM and PM collection timepoints, RNA-derived soil types at AM and PM collection timepoints), and analysis being used (**A**. Observed Richness, **B**. Shannon Diversity Indices, **C**. Simpsons Diversity Indices). Lines in the boxes indicate the median, with the top and bottom of the boxes representing the 75th and 25th quartiles. Whiskers represent the 1.5 × interquartile range (IQR). Stars indicate significant differences between derived community, soil types, and collection timepoint (NS = *p*-value > 0.05, *p-value < 0.05, **p-value < 0.01, ***p-value < 0.001, ****p-value < 0.0001).

**Figure 3:**
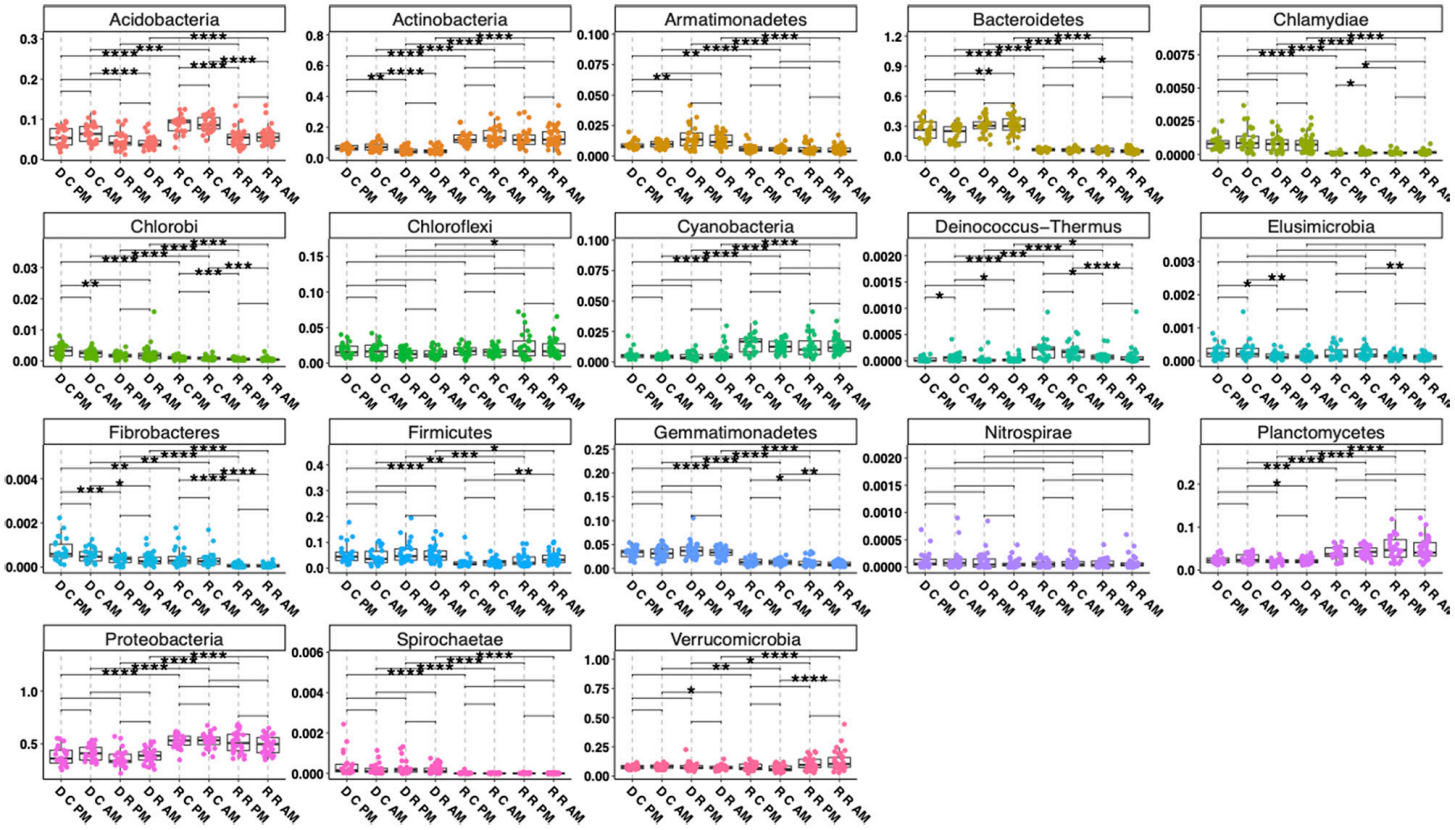
Boxplots of phylum relative abundance by sample type. Lines in the boxes indicate the median, with the top and bottom of the boxes representing the 75th and 25th quartiles. Whiskers represent the 1.5 × interquartile range (IQR), with colored points representing the relative number of reads of a phylum within an individual sample. Stars indicate significant differences between sample types by soil types within the RNA and DNA groups and by time of collection (“ ” = *p*-value > 0.05, *p-value < 0.05, **p-value < 0.01, ***p-value < 0.001, ****p-value < 0.0001). The first character on the *x*-axis represents the nucleic acid the sample was derived from (D, DNA; R, RNA), the second the soil type (C, Control Soil; R, Rhizosphere Soil), and the last two characters represent the time of collection (PM, early afternoon light; AM, pre-dawn darkness).

Using rarefied communities, observed alpha diversity significantly differed between the RNA- and DNA-derived communities in the Control AM (*p* < 0.05), Rhizosphere PM (*p* < 0.01), and Rhizosphere AM (*p* < 0.01) soils. Only the DNA control soil AM and RNA control soil AM differed in Shannon (*p* < 0.05) and Simpson (*p* < 0.01) diversity (Figure 2). This indicates that within a sample, the diversity of what bacteria are present differs from the diversity of which bacteria are contributing to the community PSP profile. Visualization of the relative abundance of phyla (Figure 3) between the RNA- and DNA-derived communities showed significant differences between the eight soil-time sample types in all but 2 (Elusimicrobia, Nitrospirae) of the 18 total number of phyla identified in this study, indicating that microbial membership and community PSP profile differed for all soil-time sample groups. Three phyla showed minimal to no differences between the RNA- and DNA-derived communities, indicating that, in terms of the number of microbial members in the community, they contribute equivalently to the community PSP profile. Chloroflexi only showed significant (^*^p < 0.05) differences between the RNA- vs. DNA-derived rhizosphere soils collected at the AM timepoint. Furthermore, Actinobacteria (DNA vs. RNA rhizosphere PM collection *p* > 0.05 NS) and Verrucomicrobia (DNA vs. RNA control soils PM collection *p* > 0.05 NS; Figure 3).

**Figure 4:**
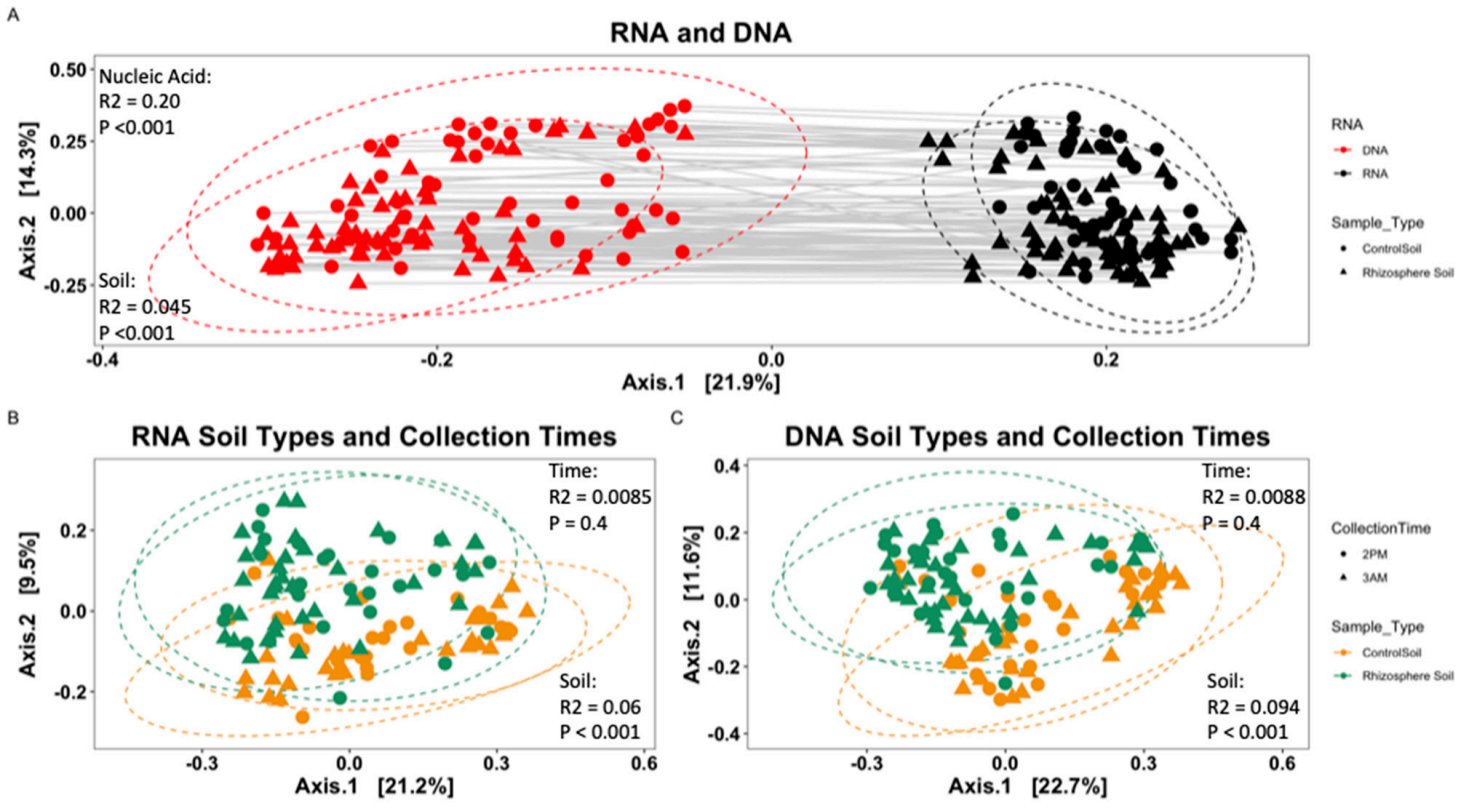
Metric multidimensional scaling (PCoA) plot of bacterial community based on Bray-Curtis dissimilarities. **(A)** RNA-derived vs. DNA-derived community. Solid gray lines connect each RNA-derived sample with its corresponding DNA sample. Shape represents soil type of origin (circle, Control Soil; triangle, Rhizosphere Soil), and color denotes nucleic acid of origin (red, RNA; black, DNA). **(B)** RNA-derived bacterial community and **(C)** DNA-derived bacterial community. All points were normalized by abundance within a sample. Points represent unique samples, with color indicating soil sample type (yellow, Control Bulk Soil; green, Rhizosphere Soil). Ellipses represent the 95% confidence interval of the mean for each soil type.

Beta diversity analysis via ANOVA model OTU abundance (within RNA- or DNA-derived community) = Soil Type ^*^ Collection Date ^*^ Field Block on the RNA- and DNA-derived communities together showed RNA (nucleic acid of origin) to be significant (^***^P < 0.001; *R*^2^ = 0.20436) and (Figure 4A), as well as soil type (rhizosphere or bulk-control; ^***^P < 0.001; *R*^2^ = 0.04486), field block (^**^P < 0.006; *R*^2^ = 0.00766), and Soil Type: Collection Date (^**^P < 0.002; *R*^2^ = 0.01342). The low *R*^2^ effect size suggests that the nucleic acid of origin has the most significant impact on microbial community composition, though even that effect is small. Other factors, such as the spatial scale of the field block or stochastic differences of collection day, had minimal to no effects.

**Figure 5:**
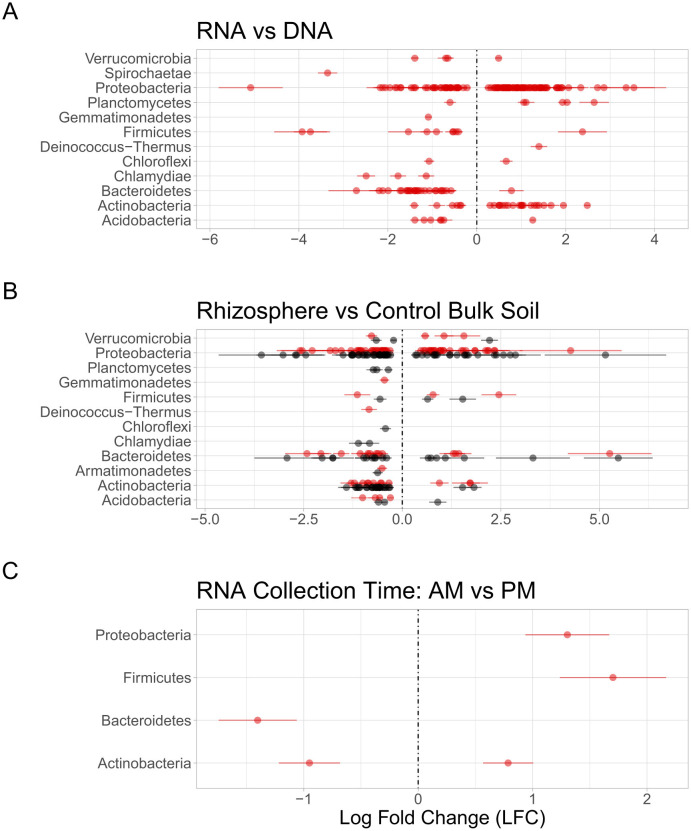
Differential abundance calculated for each genus. Each line across the graph represents a phylum, and each point a unique genus within that phylum, with lines of standard error around each point. Each genus in **(A, B)** has significantly different (*p*-value < 0.01) differential abundance between the indicated communities, with **(C)** having a significance of *p* < 0.05. **(A)** Compares abundances of RNA-derived (red) genera to DNA-derived genera, with zero being the baseline DNA. Genera above zero were more abundant in the RNA community than the DNA community, and genera below zero were less abundant in the RNA community. **(B)** Compares genera in the rhizosphere and control soils, with zero being the baseline control soil genera. Genera above zero were more abundant in the rhizosphere than in the control soils, and genera below zero were less abundant in the rhizosphere compared to control soils. Red points represent genera derived from the RNA community, and black colored points below the line represent genera derived from the DNA community. **(C)** Compares genera collected from RNA-derived rhizosphere soils between the AM and PM collection times, with the zero baseline being the AM timepoint. Genera above zero were more abundant in the PM timepoint than the AM timepoint, and genera below zero were more abundant in the AM than the PM.

Differential abundance analysis via corncob identified 12 phyla (202 genera) that differed significantly (*p* < 0.01) between DNA- and RNA-derived communities. Of these, 108 were more abundant in the RNA-derived community compared to the DNA-derived community (Figure 5A), indicating a significantly higher PSP for the number of cells present.

### Differences in community composition between soil environments (rhizosphere vs. control soils)

Using rarefied communities, alpha diversity differed significantly between the rhizosphere and control soils in the DNA-derived microbial community in the observed richness (DNA Control PM to DNA Rhizosphere PM *p* < 0.05; DNA Control AM to DNA Rhizosphere AM *p* < 0.01), Shannon (DNA Control PM to DNA Rhizosphere PM *p* < 0.01; DNA Control AM to DNA Rhizosphere AM *p* < 0.0001), and Simpsons (DNA Control PM to DNA Rhizosphere PM *p* < 0.001; DNA Control AM to DNA Rhizosphere AM *p* < 0.0001) diversity indices. No significant differences in alpha diversity were observed between the different soil types in the RNA-derived communities, except in Shannon diversity between the RNA rhizosphere and control soils collected at 3 AM (*p* < 0.5; Figure 2), indicating that while microbial membership differed within samples, the diversity of what bacteria were contributing to the PSP profile within a sample did not.

Separately analyzing the RNA- and DNA-derived communities from one another, rhizosphere and control soils differed significantly from each other (^*^p < 0.05) for 7 (Acidobacteria, Bacteroidetes, Chlorobi, Deinococcus-Thermus, Elusimicrobia, Fibrobacteres, Verrucomicrobia) of the 18 total phyla identified in this study. Three unique phyla (Actinobacteria, Armatimonadetes, Planctomycetes) showed significant (^*^p < 0.05) differences only in the DNA-derived rhizosphere and control soil microbial communities, while three unique phyla (Chlamydiae, Firmicutes, Gemmatimonadetes) showed significant (^*^p < 0.05) differences only in the RNA-derived microbial communities (Figure 3). This indicates the differences in microbial membership and microbial contribution to community PSP profile differ according to phyla within the rhizosphere and control soils. Using the model OTU abundance (within RNA- or DNA-derived community) = Soil Type ^*^ Collection Date ^*^ Field Block showed significant differences in community composition within the RNA- and DNA-derived samples. In the RNA (Figure 4B), soil type (*P* < 0.001^***^; *R*^2^ = 0.05962), field block (*P* < 0.011^**^; *R*^2^ = 0.01990), and soil type: collection date (*P* < 0.037^*^; *R*^2^ = 0.03288) were significant. In the DNA (Figure 4C) only the soil type was significant (*P* < 0.001^***^; *R*^2^ = 0.094). However, the low *R*^2^ effect size suggests a very small effect of these factors on microbial community composition.

We used the corncob Bayesian analysis to identify individual taxa that differed between the rhizosphere and control soils. In the DNA-derived community, 10 phyla (114 genera) were significantly different (*p* < 0.01) between control and rhizosphere soils. Of these, 35 genera were more abundant in the rhizosphere soil compared to the control soil community (–log10 *p*-value above zero; Figure 5B). In the RNA-derived community, corncob differential abundance analysis identified 10 phyla (96 genera) that differed significantly (*P* < 0.01) between control and rhizosphere soils. Of these, 39 genera were more abundant in the rhizosphere soil compared to the control soil community (Figure 5B). Between the DNA- and RNA-derived communities, 58 genera (7 phyla) overlapped, with 23 genera being above zero and 34 below zero. In the DNA-derived community, 57 genera were unique, with 12 having more than zero and 45 having fewer than zero. In the RNA-derived community, 39 genera were unique to the corncob analysis, with 16 genera having a positive value and 23 having a negative value.

### Differences in community composition by collection timepoint, 4 AM vs. 2 PM

Using rarefied communities, alpha diversity did not differ significantly between the two collection timepoints. Visualization of the relative abundance of phyla between the AM and PM collection timepoints showed no significant differences, except in two phyla between the DNA-derived control soils (Deinococcus-Thermus, ^*^p < 0.05), where the AM timepoint was greater, and the RNA-derived control soils (Chlamydiae, ^*^p < 0.05), where the AM timepoint was greater (Figure 3).

To remove the confounding effect of collection date, samples were separated into three groups corresponding to the date of collection, in addition to being separated by their nucleic acid type, and the model OTU abundance (within RNA- or DNA-derived community) = Soil Type ^*^ Collection Time ^*^ Field Block was used to calculate significance. Community composition was significantly different between soil type (^**^P < 0.004; *R*^2^ = 0.06336) and field block (^**^P < 0.008; *R*^2^ = 0.0542) on day one and soil type only on day 2 (^***^P < 0.001; *R*^2^ = 0.08644) and day 3 (^***^P < 0.001; *R*^2^ = 0.11179). In the DNA community, soil type (^***^P < 0.001; *R*^2^ = 0.09588) and field block: sample type (^*^P < 0.021; *R*^2^ = 0.05150) on day one, and soil type only on day 2 (^***^P < 0.001; *R*^2^ = 0.11893) and day 3 (^***^P < 0.001; *R*^2^ = 0.13241). The collection timepoint was never significantly different between the RNA- nor DNA-derived microbial communities (Figures 4B, C). However, the low *R*^2^ effect size suggests a very small effect of these factors on microbial community composition, though the effect of collection day varied from a very small to small effect.

Samples were pooled once more by collection date, then separated based on soil type and RNA- and DNA-derived community to calculate significance using the model OTU abundance (within RNA- or DNA-derived community) = ~Collection Time. Collection Time was not significant in either rhizosphere (RNA: *P* = 0.04; *R*^2^ = 0.0085; DNA: *P* = 0.04; *R*^2^ = 0.0088) or in control soils. Here again, the *R*^2^ effect size is very small, indicating that this result is insignificant.

**Figure 6:**
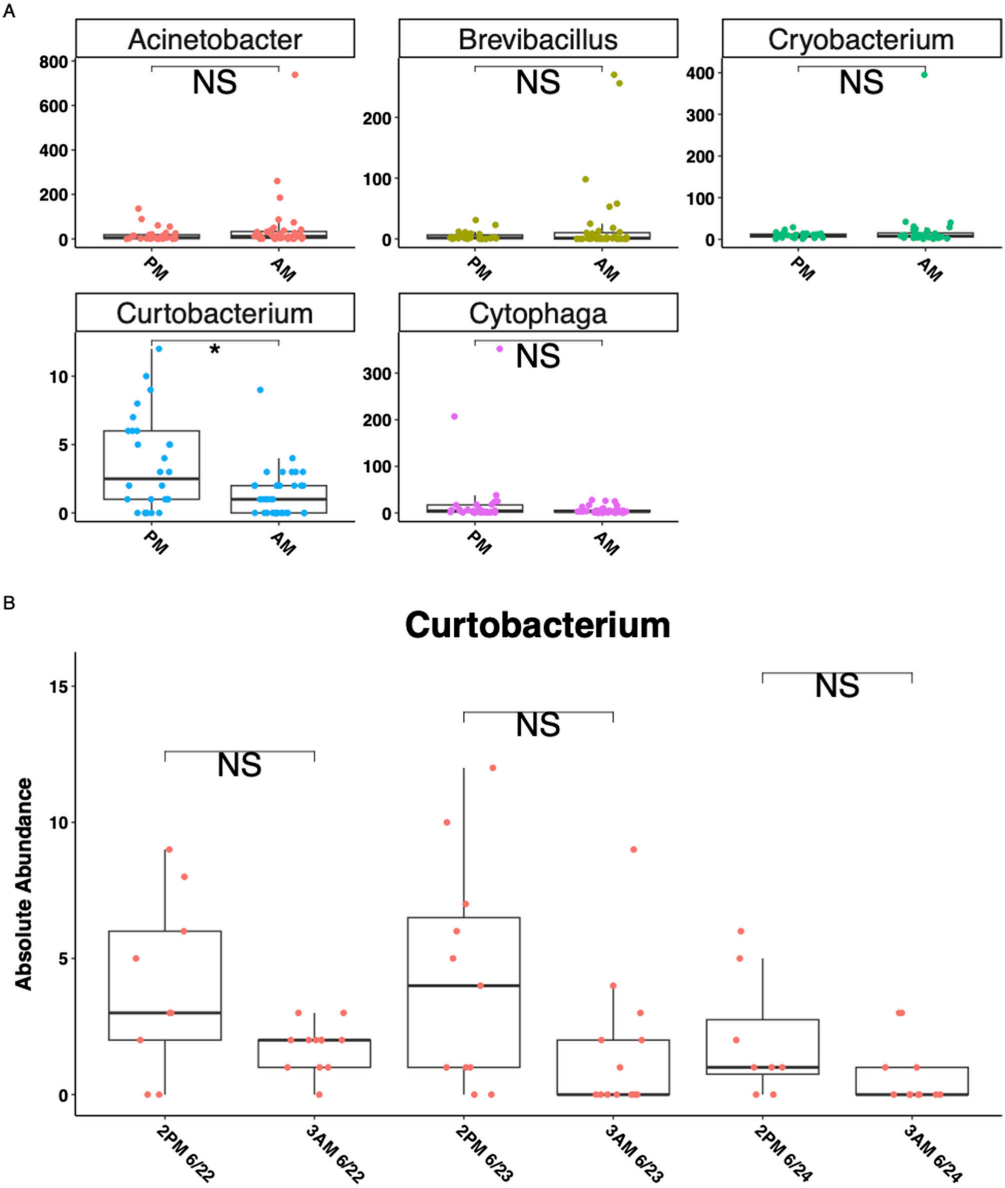
Five genera with significantly (*p* < 0.05) different absolute abundances between AM and PM timepoints in RNA-derived rhizosphere soils. *Y*-axes for all plots are absolute abundance of reads per sample, *x*-axes are collection timepoints in the early afternoon (2 PM) or pre-dawn (3 AM). **(A)** The absolute abundance of the five genera corncob differential abundance analysis identified as significantly (*P* < 0.05) different between timepoints, **(B)** separates the Curtobacterium genus by day of collection as well as time. Lines in the boxes indicate the median, with the top and bottom of the boxes representing the 75th and 25th quartiles. Whiskers represent the 1.5 × interquartile range (IQR), with colored points representing the relative number of reads of a phylum within an individual sample. Stars indicate significant differences between sample types by soil types within the RNA and DNA groups and by time of collection (“NS” = *p*-value > 0.05, *p-value < 0.05).

Corncob differential abundance analysis identified five genera that differed significantly (^*^P < 0.05) between the 3 AM and 2 PM collection timepoints of RNA-derived rhizosphere soil microbial communities (Figure 5C). No genera were significantly (^*^P < 0.05) different between the RNA-derived control soils, or the DNA-derived rhizosphere or control soils. Observing the absolute abundance of reads of the genera determined to be significantly different from the corncob analysis shows only one genus, Curtobacterium, to be significantly (^*^P < 0.05) different between collection timepoints (Figure 6A), but the amplitude of the signal was not significant when collection time was separated within collection days (Figure 6B).

## Discussion

The goal of this study was to investigate changes in soil microbial communities across short timescales under field conditions by comparing 16S-transcript (RNA) and 16S-gene (DNA) generated amplicons. Significant differences were observed between the overall RNA- and DNA-derived communities, with RNA-derived reads showing more significant differences between the rhizosphere and control soil communities within phyla and in differential abundance analyses of genera. RNA-derived communities were more sensitive to the effect of field block and collection date, suggesting differences in community activity and possibly protein synthesis potential over fine spatial scales (blocks) and over short, 24-h timescales (collection date). The community reconstructed from RNA showed significant differential abundance for five genera in the rhizosphere soil between the pre-dawn (AM) and early afternoon (PM) timepoints. However, only Curtobacterium showed a significant difference in total read numbers between timepoints, although the amplitude of the signal was not significant when separating reads based on collection days. These results suggest that there is little diel cycling among microbial communities in field settings despite their observation in controlled ones. The differences in read number and diversity observed between the overall RNA- and DNA-derived microbial communities reflect the differences between total microbial membership and protein synthesis potential (PSP) and are consistent with those observed in other studies (Baldrian et al., 2012; Bowsher et al., 2019; Gill et al., 2017; Klein et al., 2016; Li et al., 2019; Mikkonen et al., 2014; Schostag et al., 2020), as well as by previous studies on the same study system (Ceretto and Weinig, 2025).

The higher proportion of RNA-derived reads indicates a greater number of ribosomes relative to the gene-copy number within an active living cell (Baldrian et al., 2012; Moeseneder et al., 2005). In Figure 2, taxa with significantly higher DNA-derived reads compared to RNA-derived (such as those in phyla Gemmatimonadetes, Bacteriodetes, and Chlamydiae) are likely not as active in the microbial community, as DNA can include genes from dead or lysed cells, free extracellular DNA, or dormant cells with low ribosomal counts (Bakken and Frostegård, 2006; England et al., 2004; Hamilton et al., 1968; Lorenz and Wackernagel, 1987). The taxa with significantly high RNA-derived read numbers (such as those in phyla Actinobacteria, Proteobacteria, and Plantomycetes) are likely highly active participants in microbial community functions, such as soil biochemical cycles and microbial metabolism, or are responding to environmental cues that affect cellular functions (Blazewicz et al., 2013; Bulgarelli et al., 2012; Schimel and Bennett, 2004).

Observed patterns of RNA- vs. DNA-derived community reconstruction appear to be robust across growing seasons. In the same study system, for instance, we found similar differences in microbial community membership and PSP in different years (Ceretto and Weinig, 2025). Taxa between studies used the same database for taxonomic identity assignment, so comparisons of microbial taxa are equivalent. We observed similar patterns in alpha diversity, where the RNA-derived community was not significantly different between the bulk-control and rhizosphere soil, but the DNA-derived community was significantly different (Figure 2). Similar phyla were significantly different in PSP vs. microbial membership. Phyla with significantly higher RNA than DNA reads, such as Acidiobacteria, Actinobacteria, Proteobacteria, Cyanobacteria, Deinococcus-Thermus, and Planctomycetes, were found in both studies, as were phyla with significantly fewer RNA to DNA reads, such as Gemmatimonadetes and Chlamydiae (Figure 3). Comparing the overall RNA-to-DNA-derived communities between studies showed that the phyla with significantly differentially abundant taxa were the same between studies. Only Deinococcus-Thermus, with one genus higher in RNA-derived reads, and Spirochaetae, with one genus higher in DNA-derived reads (Figure 5A), were not present in our previous study. The same phyla showed significantly differentially abundant taxa when comparing the rhizosphere and bulk-control soils, with a similar distribution of significant taxa between soil types and RNA- and DNA-derived reads (Figure 5B).

That the RNA-derived community was more sensitive to fine-scale environmental changes, which may affect cellular functions and protein synthesis potential, such as field block, collection time, and soil type, is consistent with other studies (Charvet et al., 2014; De Vrieze et al., 2018; Hunt et al., 2013; Li et al., 2019). Overall community composition between the rhizosphere and control soils was similar, though the heightened PSP of some phyla in the rhizosphere compared to control soils (Figures 3, 5B) is consistent with other studies that utilize RNA-derived communities (Li et al., 2019), as well as studies that show rhizosphere soil microorganisms have heightened enzyme PSP compared to bulk soils (Jacoby et al., 2021; Ren et al., 2021).

We observed significant differences in abundances of five genera (phyla Proteobacteria, Firmicutes, Bacteroidetes, and Actinobacteria) between the pre-dawn (3 AM) and early afternoon (2 PM) timepoints in RNA-derived rhizosphere soils (Figures 5C, 6A). However, a closer examination between the 3 days of sampling did not show a consistently strong amplitude of signal between the two timepoints in any of the five genera (Figure 6A). Curtobacterium, in the phylum Actinobacteria, showed the most significant differences between the absolute number of reads in the AM and PM timepoints, showing higher PSP in the rhizosphere soils during the dark pre-dawn AM. This genus exhibited some cyclical fluctuations between the three collection days at the timepoints (Figure 6B), although these fluctuations were not significant.

The small variations observed in Curtobacterium's PSP could be due to the genus's strong association with plant physiology. Some Curtobacterium strains are commonly found in soils, where they decay complex carbohydrates found in plant leaf litter (Chase et al., 2016), while others found in the phyllosphere and rhizosphere are known plant growth promoters able to elicit plant defense responses and disease resistance to pathogens, while others are plant pathogens (Bulgari et al., 2009; Raupach and Kloepper, 1998; Schillaci et al., 2022). The fine-scale changes in a plant's physiology, such as photosynthesis-derived root exudation and water allocation, and other physiological responses associated with diel cycling (Hubbard et al., 2018; Nobs et al., 2019; Seaton et al., 2015; Zhao et al., 2021), could be driving the faint patterns observed in Curtobacterium PSP as the microorganisms increase the number of ribosomes within their cells for possible metabolization of root exudates or other small chemical changes in the rhizosphere mediated by the plant.

Other studies have shown rhizosphere microorganisms responding to plant circadian rhythms and diel cycling (Harmer, 2009; Hubbard et al., 2018; Maddur Puttaswamy, 2019; Zhao et al., 2021). In these studies, host plant circadian clock function explained up to 19% of the variance in microbial community composition. The preceding studies indeed included the study organism used here, *B. stricta* (Hubbard et al., 2023, 2018); however, they were performed in controlled growth chamber settings. It is likely that other ambient factors in the field, such as temperature fluctuations, variable sunlight, and almost certainly intermittent precipitation events, outweighed or weakened the microbial community's response to rhizosphere signaling of the host plant (Dacal et al., 2019; Evans et al., 2022; Zhao et al., 2021).

## Data Availability

The data presented in the study are deposited in the Wyoming Data Repository, accession number https://doi.org/10.15786/8VDJVB.
